# Site-level variation in field of view is associated with altered anti-predator responses in farming damselfish

**DOI:** 10.1093/beheco/araf102

**Published:** 2025-09-12

**Authors:** James S Boon, John E Stratford, Jason Lynch, Chris Yesson, Richard Field, Dan A Exton, Sally A Keith

**Affiliations:** School of Geography, University of Nottingham, Nottingham, NG7 2RD, United Kingdom; School of Natural and Environmental Sciences, Newcastle University, Newcastle upon Tyne, NE1 7RU, United Kingdom; Institute of Zoology, Zoological Society of London, Outer Circle, Regent's Park, London NW1 4RY, United Kingdom; Institute of Zoology, Zoological Society of London, Outer Circle, Regent's Park, London NW1 4RY, United Kingdom; School of Geography, University of Nottingham, Nottingham, NG7 2RD, United Kingdom; Operation Wallacea, Wallace House, Old Bolingbroke, Spilsby PE23 4EX, United Kingdom; Lancaster Environment Centre, Lancaster University, Lancaster, LA1 4YW, United Kingdom

**Keywords:** Coral reef fish, Habitat complexity, Predator avoidance, Predator–prey, Risk assessment

## Abstract

The three-dimensional (3D) structure of habitats influences how prey detect and respond to predators, but the specific roles of different aspects of structural complexity remain poorly understood, particularly in coral reef ecosystems. We used 3D models of 3 Caribbean reef sites to quantify 3 structural metrics at site level: field of view (the extent of observable area), refuge density (density of holes), and rugosity (reef surface roughness). We then observed the anti-predator behavior of damselfish, parrotfish, and wrasses at each site. Territorial damselfish showed species-specific responses to habitat structure, especially in relation to field of view. *Stegastes adustus*, for example, exhibited shorter flight initiation distances (FIDs) at the site with the highest field of view, consistent with expectations from optimal escape theory. In contrast, wrasse and parrotfish species showed little variation in behavior across sites, though larger individuals tended to have longer FIDs and flight distances. Refuge density was similar across sites, likely reflecting long-term regional loss of fine-scale complexity in the Caribbean. While rugosity is widely used as a proxy for reef complexity, our results suggest that field of view may be more strongly associated with differences in anti-predator behavior, particularly in damselfish. These findings highlight the need to assess multiple dimensions of habitat structure, as even closely related species may exhibit distinct behavioral adaptations to their 3D environment.

## Teaser

The physical structure of a habitat shapes how prey detect and escape predators, but different aspects of this structure can affect how prey perceive risk. Using three-dimensional models of three Caribbean reefs, we measured three structural features: how much area fish can see (field of view), the number of hiding places (refuge density), and the roughness of the reef surface (rugosity). We then observed the responses of damselfish, parrotfish, and wrasses to a model predator at each site. Damselfish showed clear species-specific patterns. For example, *Stegastes adustus* allowed predators to approach more closely before fleeing when visibility was greatest, consistent with the idea that prey feel safer when they can see more of their surroundings. Wrasses and parrotfish showed little variation across sites, although larger individuals tended to flee sooner and travel further. Refuge density was similar across all reefs, likely reflecting long-term degradation of Caribbean reef structures. Overall, the results suggest that field of view may influence damselfish behaviour more strongly than other aspects of structural complexity, highlighting the need to consider multiple dimensions of reef structure, as different species adapt in distinct ways.

## Introduction

The three-dimensional (3D) structure of a habitat plays an important role in shaping how species are distributed and behave (MacArthur and MacArthur 1961; McCoy and Bell 1991; Warfe and Barmuta 2004). Habitats with greater structural complexity generally support greater species abundance and diversity due to the increased availability of niches, sheltered areas, and resources (August 1983; Gratwicke and Speight 2005; Ghadiri Khanaposhtani et al. 2012; Graham and Nash 2013; St. Pierre and Kovalenko 2014). The 3D structure of a habitat is particularly important to predator–prey dynamics, as prey can assess predation risk based on predator visibility, available cover, and escape options (Warfe and Barmuta 2004; Camp et al. 2013).

Optimal escape theory states that the escape responses of prey are influenced by a tradeoff between the perceived risk of predation and the energetic cost of abandoning activities, such as foraging or mating, to engage in an escape response (Ydenberg and Dill 1986; Cooper and Frederick 2007). This decision-making process can be influenced by environmental characteristics, such as the availability of crevices or shelters that provide refuge from predators (Berryman and Hawkins 2006). When refuges are sparse and far away, an individual's perceived risk of predation is thought to increase due to the higher energetic costs required to reach a safe area (Stankowich and Blumstein 2005). Increased distance to the nearest refuge is associated with heightened risk aversion across several taxa, including birds (Morelli et al. 2022), mammals (Dill and Houtman 1989), fish (Dill 1990), and reptiles (Cooper 2007). Likewise, an individual's field of view (ie, extent of observable area from a given position), which can be shaped by the topography of their environment, is also thought to affect when individuals begin to assess predation risk (Ndaimani et al. 2013; Stein et al. 2022). A wider field of view allows for earlier predator detection, while a limited field of view delays predator detection until they are closer (Embar et al. 2011; Mols et al. 2022; Gresham et al. 2023). According to the “flush early and avoid the rush” hypothesis, animals flee shortly after detecting a threat, thereby minimizing the costs associated with continued vigilance (Blumstein 2010). Evidence supporting this hypothesis has been observed in birds and mammals, but it appears to be less applicable to other taxa, such as lizards (Samia et al. 2013).

In reality, perceived risk is influenced by an interaction among these various aspects of structural complexity, further mediated by biological factors. For instance, red deer (*Cervus elaphus*) displayed greater risk aversion in areas with very high and very low fields of view, instead having a preference for habitats offering an intermediate level of complexity (Zong et al. 2023). This preference likely comes from a tradeoff, as deer require some degree of complexity for concealment to reduce predation risk, they also need open views to detect predators (Zong et al. 2023). Body size can have further confounding effects on this relationship (Chan et al. 2019). According to the asset-protection principle, larger individuals, possessing greater energy reserves, can afford to prioritize safety in environments with many refuges, while smaller individuals may need to forage more frequently and accept higher risks due to limited resources (Wahle 1992). For example, larger sticklebacks (*Gasterosteus aculeatus*) prioritize safety over feeding more so than smaller individuals due to the relatively lower energy costs associated with fleeing, but in areas with fewer refuges, even larger individuals need to risk predation to fulfill their dietary needs (Krause et al. 2000). Defense strategies, such as crypsis, can also influence predator-avoidance behaviors (Samia et al. 2016). Species that rely on crypsis are more likely to freeze rather than flee (Samia et al. 2016), and in structurally complex environments, this stillness may further reduce detection by predators. This underscores the interactive effects that different structural features and biological factors can have on anti-predator responses of prey, something which is underexplored for individuals in highly complex habitats, such as coral reefs.

The 3D structure of a reef is mainly made up of hard and soft corals, sponges, geomorphological features, and the remaining structures of dead corals (Graham and Nash 2013). When exploring how the anti-predator responses of reef fish are influenced by structure, studies often measure rugosity, a metric that assesses reef surface roughness (Luckhurst and Luckhurst 1978; González-Rivero et al. 2017). Higher rugosity, which indicates greater structural complexity, has been associated with shorter flight initiation distances (FID) of reef fish, which is the distance at which prey flee from an approaching threat (Ydenberg and Dill 1986). This pattern has been observed in several species of damselfish (Quadros et al. 2019), wrasse and parrotfish (Nunes et al. 2015), suggesting that fish in more complex habitats may perceive a lower risk of predation. Moreover, most studies use only FID to measure escape behavior, even though another valuable but underused metric is distance fled, which is the distance an individual travels after initiating flight and reflects the energy invested in escape (Ydenberg and Dill 1986; Cooper and Blumstein 2015). While used in terrestrial studies of birds (Tätte et al. 2018) and lizards (Samia et al. 2016), distance fled remains largely unexplored in reef fish. Furthermore, the relationship between reef structure and anti-predator behavior is often assessed at small spatial scales, such as individual damselfish territories (Quadros et al. 2019), potentially overlooking broader site-level characteristics and specific structural features that influence escape decisions and fish behavior (González-Rivero et al. 2017). Recent advances in underwater photogrammetry now allow for more detailed, site-level assessments of reef structure (González-Rivero et al. 2017), providing new opportunities to better understand how specific structural features influence predator–prey interactions.

This study aims to determine how the anti-predator responses of a variety of reef fish are influenced by different features of habitat complexity across 3 Caribbean reef sites of differing structural complexity. First, drawing on the “flush early and avoid the rush” hypothesis (Blumstein 2010), we predict that in more visually open environments (ie, greater field of view), fish will (1) exhibit longer FIDs, as predators can be detected earlier, and (2) flee shorter distances due to reduced perceived risk once escape is initiated. Second, based on optimal escape theory (Ydenberg and Dill 1986; Cooper and Frederick 2007), we expect that in habitats with greater refuge availability, individuals will perceive the costs of fleeing to outweigh the risks of predation (Stankowich and Blumstein 2005). Therefore, we predict that fish in areas with more refuges will (3) exhibit shorter FIDs and (4) flee shorter distances. While rugosity does not directly measure features like refuge density or visual fields, it does reflect the overall structural complexity of the habitat. We therefor hypothesize that (5) in areas with higher rugosity, fish will have shorter FIDs, as generally complex environments will lower perceived predation risk.

## Methods

### Study area

**Fig. 1.**
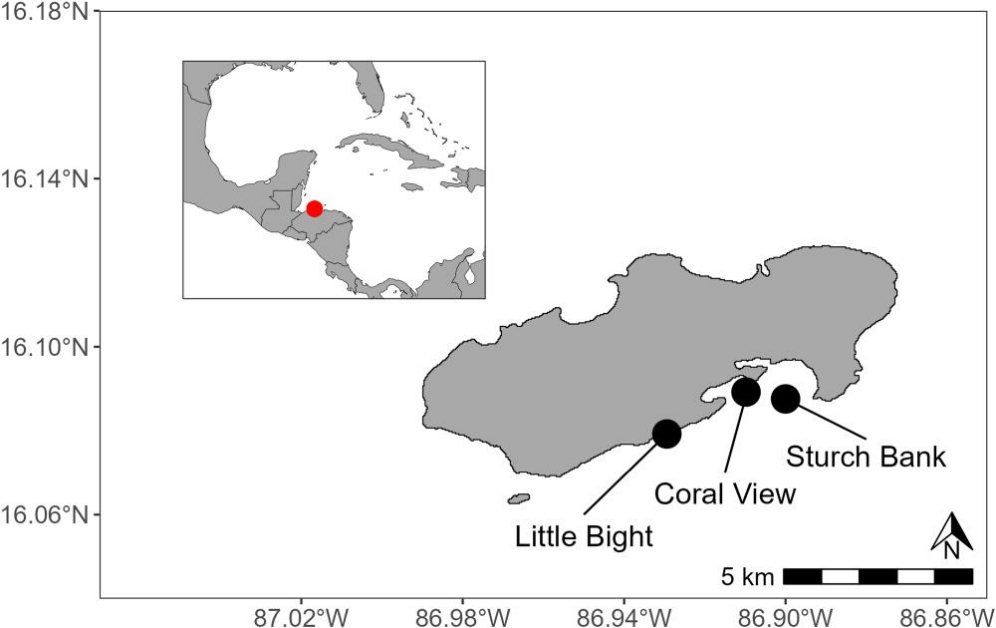
Study locations around the island of Utila, Honduras. Inset map shows the location of Utila relative to the Caribbean region. Map sourced from GADM database of Global Administrative Areas (2015).

Data were collected at 3 fringing reef sites surrounding the island of Utila, Honduras (Fig. 1). Located on the southern edge of the Mesoamerican barrier reef, Utila is a popular tourist destination, centered around the SCUBA diving industry. Sites were situated on the island's sheltered southern coastline to facilitate access and were chosen based on a priori assumptions of differing structural complexity determined by preliminary visual assessments. All sites were separated by more than 800 m. Data were collected at 5 m depth using open-circuit SCUBA. All 3 sites are shore reefs with a spur-and-groove system. While community composition was not formally assessed in this study, previous work on Utila indicates that sites along the southern shore have similar fish taxonomic and trophic compositions, with low densities of invasive lionfish (*Pterois volitans*) and piscivores at 5 m depth (Andradi-Brown et al. 2016, 2017).

### Three-dimensional (3D) reconstruction of the reef structure

We used structure-from-motion photogrammetry to model the benthic structure at Coral View, Little Bight, and Sturch Bank. The 3D reconstructions were conducted along the reef at each site, extending up to 150 m east or west of the site buoy. At each site, a 50 m transect tape was laid out at a depth of 5 m, with four 0.2-m scale markers placed at regular intervals. A diver swam 1 m above the reef, filming the benthos using 3 GoPro HERO3 cameras mounted on a straight pole at 0.5 m intervals to ensure effective image overlap. The cameras were angled 45° downward, capturing a 2-m wide area (1 m on either side of the transect tape). To optimize model reconstruction and reduce computational challenges, each 50 m × 2 m survey was divided into two 25 m × 2 m sections. In total, 12 reef transects of 25 m × 2 m were recorded at each site.

Video files from each camera were converted into images by extracting 3 frames per second using Free Video to JPG Converter v5.0.101. These images were then imported to Agisoft Metashape Professional Edition (AgiSoft 2022) and orthomosaics rendered following the protocol outlined in Young et al. (2017). Orthomosaics were then converted into point clouds, scaled and rasterized into digital elevation models (DEMs) in CloudCompare v2.11.3 (CloudCompare 2022). Resolution was ∼ 3 cm per pixel. For each of the 3 reef sites, 12 separate reconstructions were made along a 25 m × 2 m section, resulting in a total of 600 m^2^ of reef reconstructed for each site, though not in one contiguous area. All reconstructions were made between June and August 2022 by J.E.S. (see Fig. S1 for representative reconstructions from each site).

### Features of structural complexity

**Fig. 2.**
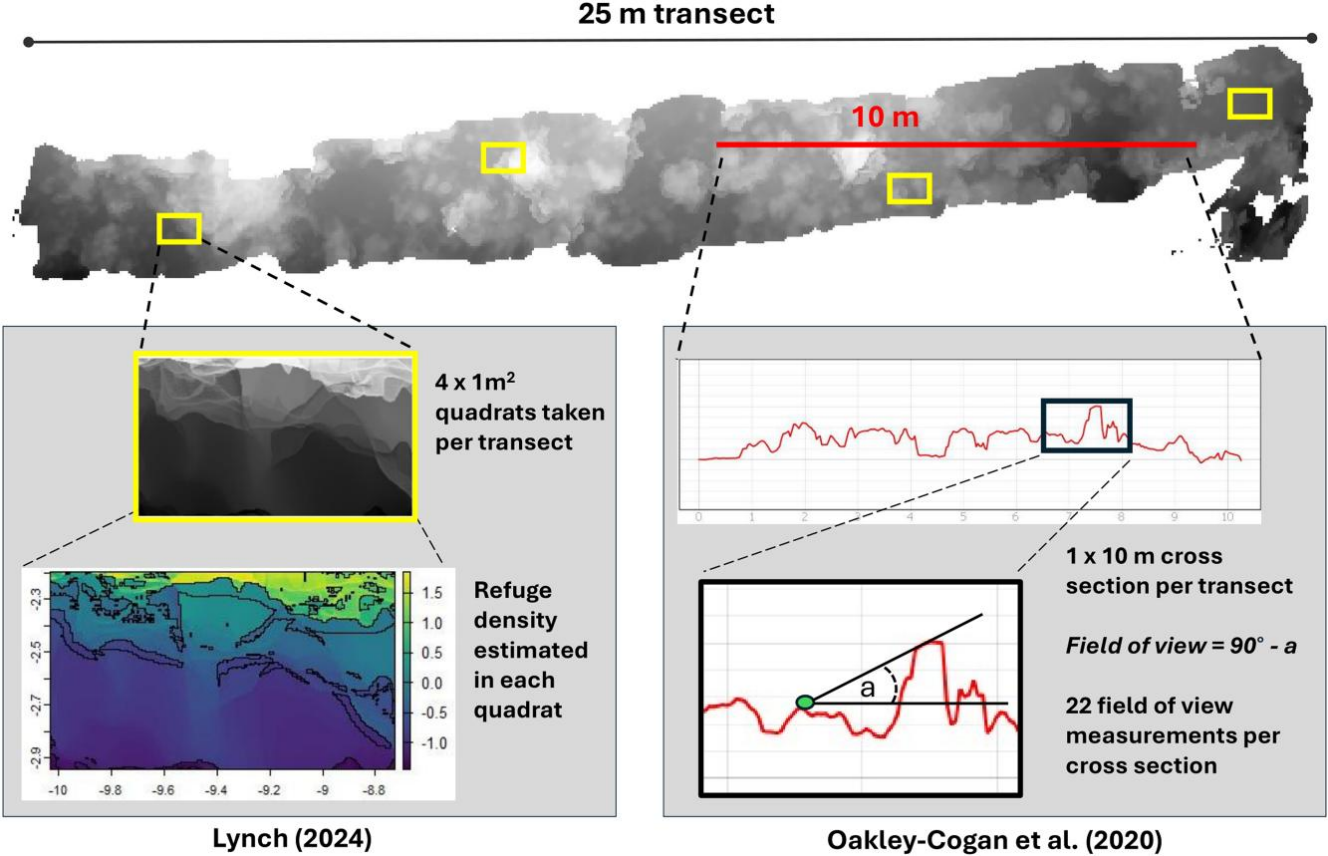
Schematic illustrating the process for sampling refuge density and field of view at each site. In each 25 m transect reconstruction (12 per site), refuge density was calculated following Lynch (2024), and field of view was determined following Oakley-Cogan et al. (2020).

Field of view was estimated following the protocol outlined by Oakley-Cogan et al. (2020). In summary, a 10-m-long cross-section was randomly generated for each 25-m segment of the DEM using the Terrain Profile tool in QGIS Desktop v. 3.20.3 (QGIS.org 2021). These cross-sections were imported and scaled in ImageJ (Schneider et al. 2012). At the start of the cross-section (0 m), a 1.8-m horizontal line was drawn towards the center of the transect, positioned 2 cm above the substrate surface to represent fish eye height. While we did not measure average eye height in our study, this value was taken from Oakley-Cogan et al. (2020) and is a reasonable approximation for our study taxa. The length of the visual line was selected as 1.8 m based on it being the average starting distance in the anti-predator experiments. An additional 1.8 m line was extended from the start of the horizontal line to the highest topographic point the angled line could reach within the cross-section. The angle formed by the horizontal line and the line to the highest elevation point was subtracted from 90 degrees, which provided the field of view (see Fig. 2 for schematic). This process was repeated at 0.5 m intervals along the cross-section, with the horizontal line always aimed towards the center of the cross-section. For the central point, lines were drawn in both directions. In each 10-m cross-section, 22 measurements were recorded and averaged, resulting in 12 values per site (one from each 25 m × 2 m DEM). Higher field of view values correspond to more open lines of sight and reflect lower complexity.

To determine the density of refuges (ie, holes on the reef) at each site, we utilized the “Hidey Hole” function (see https://github.com/cyesson/HideyHole; Lynch 2024). From the DEMs of each 25 m × 2 m transect, four 1 m² quadrats were chosen randomly and cropped (see Fig. 2 for schematic). This approach was chosen to avoid inaccuracies due to edge irregularities in the DEMs and to reduce computational demand. The function analyzed each cropped quadrat by examining each pixel's elevation relative to its surrounding neighborhood to identify depressions. It calculates a local average elevation and flags pixels significantly lower than this average, using a user-defined depth threshold (Dagum et al. 2021). Identified pixels were grouped into contiguous polygons representing potential hidey holes. Here, we used a depth threshold of 5, 10, and 15 cm, respectively. These hole depths were chosen based on the average length of taxa assessed in this study, with the assumption that any larger holes would not provide a sufficient predation shelter. We estimated the total count of 5-, 10-, and 15-cm holes per 1 m^2^ across sites. Higher hole densities correspond to more potential refuge opportunities.

Planar rugosity was calculated by dividing the geometric surface area of each DEM by the true surface area and then subtracting that value from one (Young et al. 2017). For each site, we obtained 12 rugosity measurements (one from each 25 m × 2 m DEM), where values ranged from zero to one, with higher values correspond to greater structural complexity.

### Anti-predator responses

Predator-response experiments were conducted from 26 June to 27 July 2023. The experiments were conducted at the same site as the 3D transects, either east or west of the site buoy, extending up to 150 m in each direction. No experiments were conducted beyond this range, ensuring consistency in location where reconstructions were made. All experiments were conducted by 2 observers (J.S.B. and J.E.S.) following a standardized protocol to ensure consistency in data collection.

Experiments were conducted on a total of 10 species of reef fish across 3 taxa: wrasse (*Halichoeres garnoti* and *Halichoeres maculipinna*), parrotfish (*Scarus iseri, Scarus taeniopterus, Sparisoma aurofrenatum,* and *Sparisoma viride*), and territorial farming damselfish (*Stegastes adustus, Stegastes diencaeus, Stegastes paritus,* and *Stegastes planifrons*). These species were chosen because they were common across all study sites and are considered prey species.

Observers swam slowly around each site to identify focal taxa that were either feeding or swimming normally, and in a location that meant they could be approached horizontally. Before approaching, observers noted the species, visually estimated body size (total length in cm), determined the life stage (adult or juvenile), and, when in a monospecific group, counted the number of individuals. To minimize observer effects, both observers used identical measuring devices and practiced estimating fish lengths using plastic pipes of various sizes underwater until they consistently fell within a 1-cm margin of the actual length. Fish length estimates were practiced and revalidated every 2 to 5 d. For this study, a “group” was defined as all individuals of the same species within a 1 m radius of the focal fish (Nunes et al. 2015). Group size was accounted for because the perception of safety is thought to increase with group size (Ydenberg and Dill 1986) and influence escape behaviors (Samia et al. 2019).

An anti-predator response was initiated by moving a 3D printed and painted replica of a black grouper (*Mycteroperca bonaci*; 45 cm total length; Fig. S2) mounted on the end of a 1-m stick (to maintain observer distance) towards the focal subject. This model predator, as opposed to a diver, was used to generate a more realistic anti-predator response. *M. bonaci* was selected as previous fish community surveys around the island have shown *M. bonaci* to be present, though rare, around the island and to prey on a wide variety of fish taxa (Freitas et al. 2017). Due to the size of the model predator, individuals ≥25 cm were not included, as it was unlikely that individuals of this size would be considered prey.

All anti-predator-response experiments began with the observer positioned between 0.7 and 3 m from the focal individual at depths of ∼5 m. The observer placed a marker on the reef substrate directly beneath where the nose of the model predator was immediately before starting the experiment. The predator was then pushed horizontally towards the focal fish at a constant speed of an estimated 1 m/s. The escape response was determined to have happened when the focal individual's swimming speed surpassed the approach speed of the model predator (Januchowski-Hartley et al. 2011, 2012).

After the individual's escape, the diver placed 2 more markers to indicate the location of both the nose of the predator model and the position of the focal prey individual at the moment escape was initiated (Januchowski-Hartley et al. 2011). The planar distance (cm) between the first and third marker was measured using a measuring tape and represents the starting distance, which was recorded because the starting distance can influence anti-predator responses (Blumstein 2003). The planar distance (cm) between the second and third markers represents the FID. Escape responses were categorized as either: “fled into open water”, where fish fled but not into a shelter; “fled into refuge,” where the fish entered a hole; “evade”, where the fish maneuvered side to side or in and out of the reef structure; “none”, where no visible escape response was observed (adapted from Nunes et al. 2015). If the individual fled into open water or a refuge, a fourth marker was then dropped at the approximate location where the fish stopped fleeing (defined as when the focal individual's swimming speed dropped below that of the model predators) or at the shelter it took refuge in. The planar distance between the third and fourth marker represents the distance fled into open water or distance to refuge, depending on the escape response. A shelter was considered occupied if a fish was at least partially inside it immediately after fleeing from the model predator. Each flight experiment was conducted at least 5 m away from the previous one to minimize the likelihood of sampling the same individuals.

### Statistical analysis

Data analysis was performed in R v. 4.2.3 (R Core Team 2023). A One-Way Analysis of Variance (ANOVA) was used to determine whether there were significant differences in the mean lengths of individuals across different sites. Levene's Test was conducted to assess whether the variances in the field of view, rugosity, and refuge density were equal across sites. Since the assumption of equal variances was not required, a Welch's ANOVA was used to analyze the mean values of field of view, rugosity, and refuge density at each site. For post hoc pairwise comparisons between sites, the Games–Howell test, which is appropriate for data with unequal variances, was applied.

If differences in complexity metrics between sites were found, Bayesian mixed-effects models were then used to determine the effects of complexity on anti-predator responses using the package brm (Bürkner 2018) implemented in STAN (Stan Development Team 2023). We structured the model with one of the anti-predator behaviors (FID, distance fled, or distance to refuge) as the response variable and the interaction between species and site as a fixed effect. We acknowledge that using the site as a whole creates a spatial disconnect between the exact location of the behavioral experiments and the complexity measurement. However, our goal was to assess how broader-scale complexity at each site influences predator-avoidance behaviors. As body length and group size are known to influence escape decisions, we included these as covariates in the model. To facilitate interpretation of fixed effects, we standardized continuous covariates prior to analysis so that they had a mean of 0 and a standard deviation of 1. Models included investigator ID (J.S.B. or J.E.S.) and starting distance as random effects to account for variability in measurements between investigators and the known influence of starting distance on anti-predator responses. Models were run separately for wrasse, damselfish, and parrotfish.

We also explored how field of view and refuge density varied with rugosity. To do this, we used Bayesian linear regression with the brms package. In each model, the transect-level average of either field of view or refuge density (10-cm holes) was the response variable, and rugosity was included as a fixed effect.

All models were run with 4 chains with 3,000 iterations (1,000 warmup) using weakly informative priors (mean of 0 and standard deviation of 10) and fitted with Gaussian error distributions. We assessed model convergence through posterior predictive checks, trace plots, and ensuring that R-hat values were equal to one. All models had R-hat values of 1.00 and effective sample sizes over 1,000, demonstrating models converged well. We interpreted an effect estimate as significant if the 89% credible intervals (CrIs) did not overlap with zero (McElreath 2016). Post hoc analyses were conducted using the emmeans package in R to assess the difference in behavioral responses across structural complexity gradients (Lenth 2024). We report estimates of posterior means and emmeans contrasts, with 89% CrIs.

### Ethics statement

The study did not involve the capture or handling of fishes, only their brief disturbance when initiating an escape response. Procedures were approved by the University of Nottingham Ethics Panel and field permits were issued by the Instituto de Conservacion Forestal, Honduras (permit number: DE-MP-108-2023).

## Results

### Structural complexity metrics across sites

**Fig. 3.**
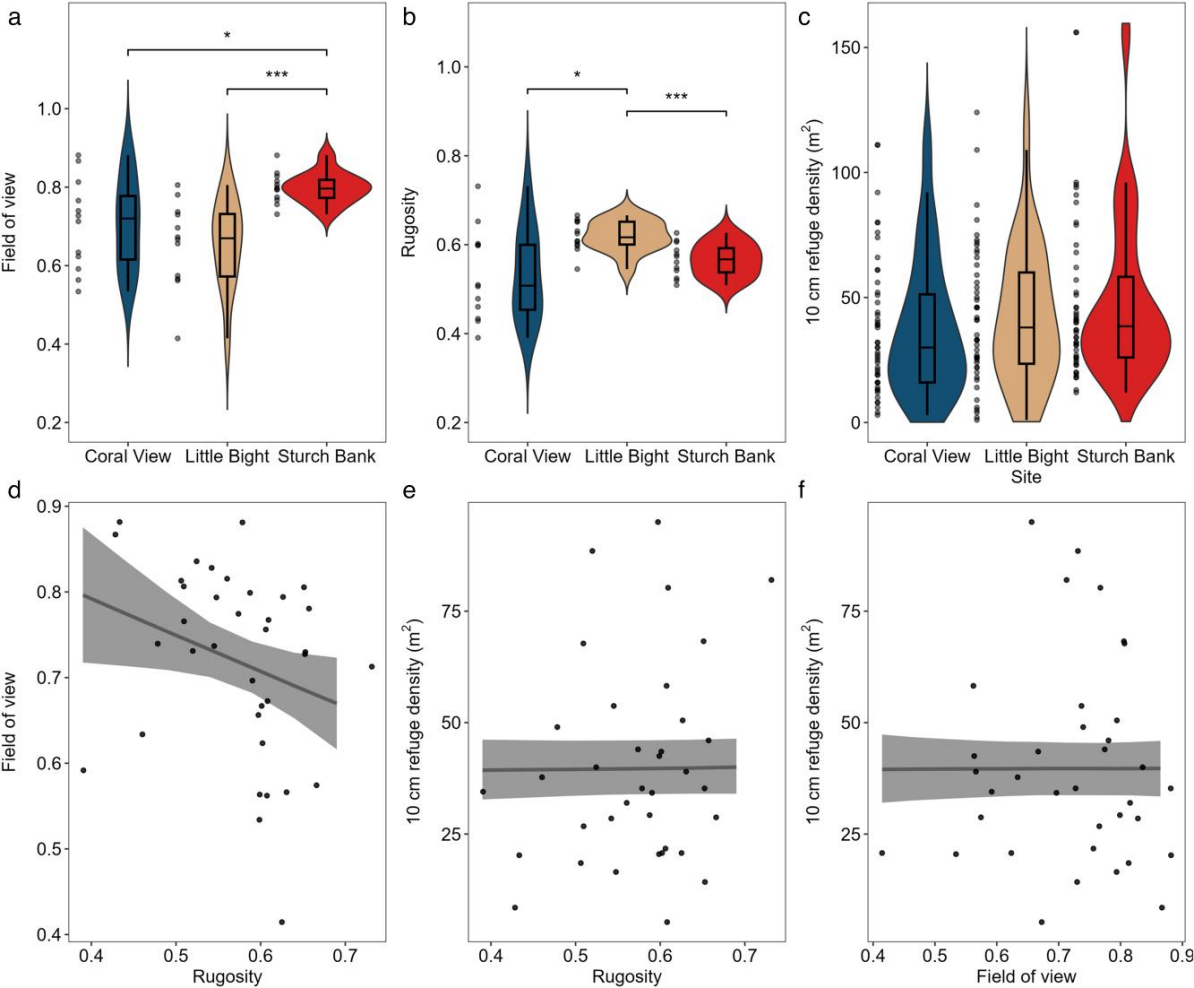
Values for (a) field of view, (b) rugosity, and (c) 10 cm refuge density across sites. Violin plots illustrate the distribution of raw values, with box plots showing the median, interquartile range, and 1.5× interquartile range (Welch's ANOVA statistical significance thresholds: “***’ = *P* < 0.01 and “*’ = *P* < 0.05). The relationship between (d) rugosity and field of view, (e) rugosity and 10 cm refuge density, and (f) field of view and 10-cm refuge density (line represents conditional effect and shading indicates 89% CrIs). Points represent raw data.

Field of view varied significantly between study sites (Welch's ANOVA, *F*_2,17.6_ = 10.99 *P* < 0.01; Fig. 3a). Sturch Bank had a greater field of view than Little Bight (0.80 ± 0.04 vs. 0.66 ± 0.11, mean ± SD) and Coral View (0.70 ± 0.12), with both differences being statistically significant (Games–Howell post hoc, *P* < 0.01 and *P* = 0.048, respectively; Levene's test, F_1,22_ = 5.79, *P* = 0.03 and *F*_1,22_ = 11.01, *P* < 0.01). No significant difference in field of view was found between Little Bight and Coral View (Games–Howell post hoc, *P* = 0.55; Levene's test, *F*_1,22_ = 0.22, *P* = 0.65).

Rugosity also varied significantly across sites (Welch's ANOVA, *F*_2,20.23_ = 8.35, *P* < 0.01; Fig. 3b). Rugosity at Little Bight was greater than at Coral View (0.62 ± 0.03 vs. 0.53 ± 0.10; Games–Howell post hoc, *P* = 0.04; Levene's, *F*_1,22_ = 8.62, *P* < 0.01) and Sturch Bank (0.57 ± 0.04; Games–Howell post hoc, *P* < 0.01; Levene's, *F*_1,22_ = 0.22, *P* = 0.64). However, no significant difference was found between Sturch Bank and Coral View.

Refuge density showed no clear differences between sites (Fig. 3c). Sturch Bank had the highest density of 10-cm deep refuges (48.04 refuges/m^2^ ± 32.50), followed by Little Bight (42.25 refuges/m^2^ ± 27.59) and Coral View (37.44 refuges/m^2^ ± 27.29), though these differences were not significant, and all showed high levels of variation (Welch's ANOVA, F_2,93.47_ = 1.49, *P* = 0.23). Likewise, there were no significant differences in the densities of 5 cm (Welch's ANOVA, *F*_2,93.56_ = 0.77, *P* = 0.46) or 15-cm refuges (Welch's ANOVA, F_2,92.92_ = 1.54, *P* = 0.22; Fig. S3). The lack of significant differences in refuge densities meant that refuge density was not modeled against anti-predator behaviors. There was a weakly negative association between transect rugosity and field of view (β = −0.42; 89% CrIs = −0.82 to −0.02; Fig. 3d) and no clear association between refuge density of 10 cm depth and rugosity or field of view (CrIs overlapped 0; Fig. 3e and f).

### Anti-predator experiments

The anti-predator responses of 389 individual fish were assessed across the 3 sites. Most parrotfish (98.5%) and wrasse (93.9%) were juveniles, whereas most damselfish (96.6%) were adults. There were no clear differences in body size within species across sites (One-Way ANOVAs, *P* > 0.10). The general response to the model predator was consistent across sites; damselfish primarily fled into a refuge (65.1%), whereas parrotfish (80.3%) and wrasse (86.6%) mostly fled into open water (Fig. S4).

**Fig. 4.**
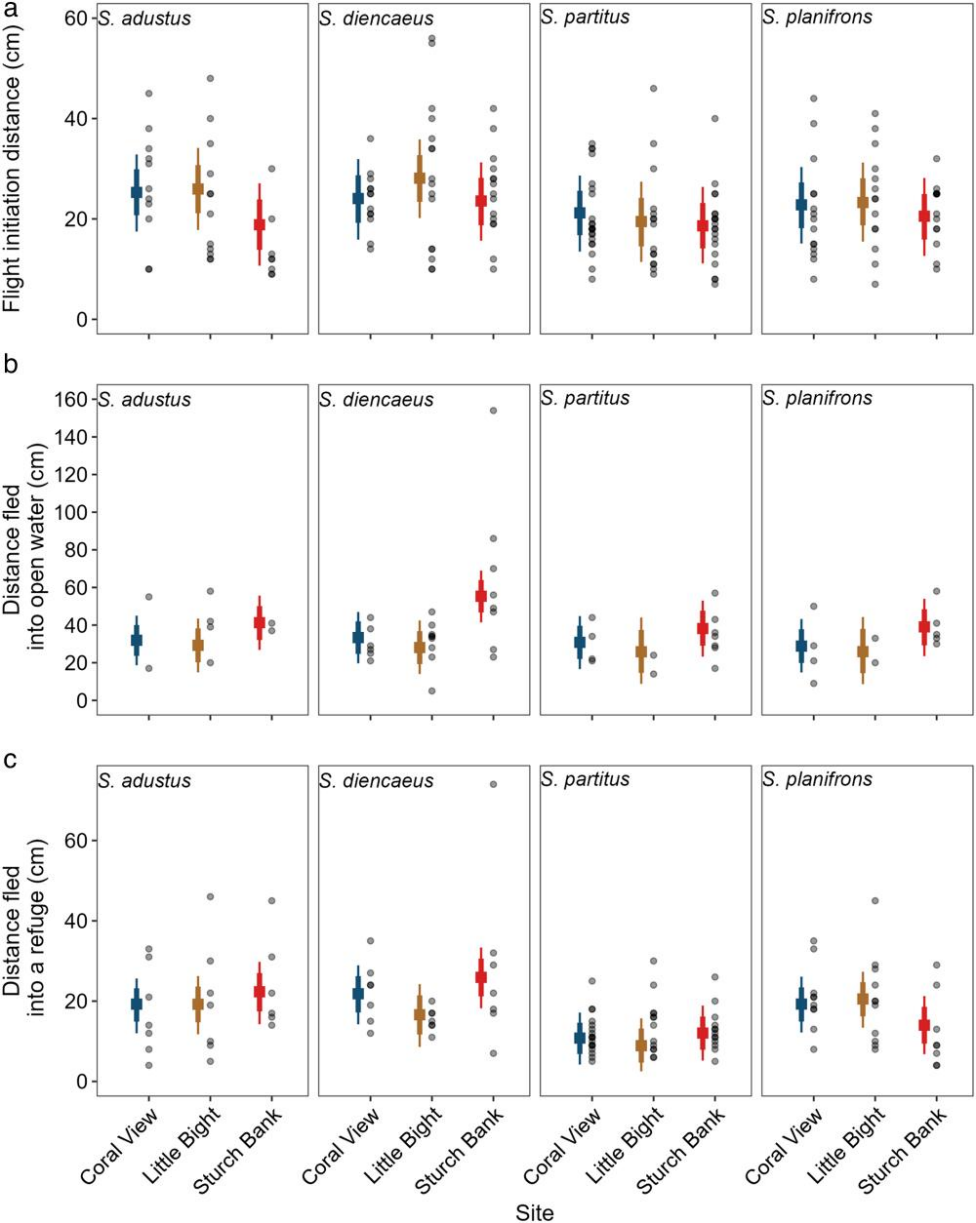
Escape responses of damselfish species across the 3 study sites. (a) Flight initiation distance, (b) distance fled into open water, and (c) distance fled into a refuge. Squares represent median estimates from Bayesian models using mean values of body length and group size. Lines show 89 and 70% highest posterior density intervals (HPDIs). Points represent raw data.

The anti-predator responses of damselfish varied across sites and species, whereas those of wrasse and parrotfish remained consistent (CrIs overlapped 0). There was strong evidence that at Sturch Bank, *S. adustus* had shorter FID compared to Coral View and Little Bight (Fig. 4a). The FID difference between Coral View and Sturch Bank was 6.46 cm (89% highest posterior density intervals (HPDIs): 0.95 cm to 11.49 cm), equivalent to 79.46% of the species' average size. Similarly, the difference between Sturch Bank and Little Bight was 7.15 cm (89% HPDIs: 0.98 cm to 12.85 cm), representing 97.95% of the species' average size. In contrast, no clear evidence suggested that FID differed between sites for other damselfish species, nor did body length or group size significantly influence FID in any damselfish species (CrIs overlapped 0). Irrespective of site, larger wrasse and parrotfish had larger FIDs (wrasse: β = 1.07, 89% CrIs = 0.00 to 3.45; parrotfish: β = 3.91, 89% CrIs = 1.61 to 6.19).

There was evidence that *S. diencaeus* fled further into open water at Sturch Bank compared to the other 2 sites (Fig. 4b). At Sturch Bank, *S. diencaeus* fled an estimated 27.43 cm farther than at Little Bight and 21.96 cm farther than at Coral View (89% HPDIs: 13.74 cm to 40.87 cm and 9.23 cm to 33.82 cm, respectively). These distances correspond to 326.16% and 261.12% of the species' average body size. In contrast, there was no clear evidence of differences in open water escape distances across sites for any other species (CrIs overlapped 0). Larger parrotfish, however, consistently fled farther regardless of site or species (β = 8.04, 89% CrIs: 1.93 to 14.02).

When fleeing into a refuge, damselfish responses were mixed (Fig. 4c). There was strong evidence that *S. diencaeus* fled farther at Sturch Bank than at Little Bight (estimated difference = 9.41 cm, 89% HPDIs: 1.74 cm to 16.81 cm; Fig. 4c), while *S. planifrons* fled shorter distances (estimated difference = −6.64 cm, 89% HPDIs: −13.42 cm to −0.53 cm).

## Discussion

Habitat structure is undergoing widespread change across multiple ecosystems (Ferrari et al. 2016; Ehbrecht et al. 2021), so understanding how behavior is influenced by structural features is important for predicting the potential impact of these changes. Our findings indicate that territorial damselfish on coral reefs may adjust their anti-predator behavior based on site-level habitat complexity, particularly field of view and rugosity. For instance, *S. adustus* exhibited shorter flight initiation distances (FIDs) at Sturch Bank, where field of view was highest, while fleeing distances varied among damselfish species across sites, highlighting species-specific differences even among closely related species. Wrasses and parrotfishes showed little variation across sites, though larger individuals consistently had longer FIDs and flight distances. These findings suggest that anti-predator behavioral theories are not universally applicable and highlight the importance of using multiple structural metrics to understand how specific habitat features shape predator-avoidance strategies.

Structural complexity varied among sites, but in general, it was dominated by large-scale rather than fine-scale features. Refuge density (ie, the number of holes within a reef) was consistent across all 3 sites. This likely reflects a long-term regional decline in fine-scale structural complexity on Caribbean coral reefs, driven by repeated stressors such as mass coral bleaching events, the loss of algal grazers, coral diseases, and increasingly frequent and intense storms (Alvarez-Filip et al. 2009, 2011b). Much of the remaining hard structural complexity is now provided by slow-growing massive coral species like *Montastrea* spp., smaller opportunistic species like Porites spp., and the eroded skeletons of dead corals (Alvarez-Filip et al. 2011a). However, these corals contribute less to fine-scale structure than the once-dominant reef-builders such as *Acropora* spp., which have declined throughout the Caribbean (Alvarez-Filip et al. 2011a; Perry et al. 2015). The reduction in refuge spaces may impact fish that rely on them for protection from predators, potentially lowering their survival and altering community structure (Rogers et al. 2014). Despite the loss of fine-scale complexity, larger features, such as geological formations and the remains of large coral colonies, remain present and continue to vary between sites, influencing the field of view, rugosity, and overall habitat complexity.

The territorial farming damselfish *S. adustus* had shorter FIDs at Sturch Bank, where the field of view was significantly greater than at other sites. This result does not align with the “flush early and avoid the rush” hypothesis, which predicts longer FIDs in more open environments where threats can be seen earlier (Blumstein 2010). Yet, this behavior does align closely with optimal escape theory, as a greater field of view would allow *S. adustus* to detect predators earlier, reducing perceived risk and therefore the need for early flight. As a species that invests heavily in maintaining and defending turf algae patches against competitors, their primary food source, *S. adustus* likely balances predator avoidance with resource protection (McDougall and Kramer 2007; Sheppard et al. 2024). Fleeing too soon after detecting a predator could lead to resource loss, so delaying escape may be an adaptive strategy to minimize this risk (Samia et al. 2013). It is unclear why only *S. adustus* showed site-based FID differences, since other territorial species like *S. diencaeus* and *S. planifrons* also defend algal patches. Although *S. adustus* are generally larger than the other species, there was no strong evidence that size influenced FID of damselfish, possibly because their secure food supply reduces foraging pressure even for smaller individuals (Wahle 1992). One possible explanation is that *S. adustus* eggs are more concealed compared to those of species like *S. diencaeus*, whose eggs are naturally more exposed (Little et al. 2013). In areas with greater visibility, they can detect predators earlier and may not need to flee as quickly, allowing them to remain near their eggs longer for protection.

Escape distances also varied among species and sites. At Sturch Bank, *S. diencaeus* invested more in their escape and fled further than at other sites, which suggests that the increased field of view may heighten their perception of predation risk in more open environments. Once an escape response is initiated, individuals may perceive a greater risk in open water due to increased visibility to predators, meaning they extend their fleeing distance to avoid exposure and reach a safer location or distance. In contrast, when fleeing into a refuge, *S. planifrons* fled shorter distances at Sturch Bank. This could be because the greater field of view allowed *S. planifrons* to detect predators earlier, so it began to move closer to its refuge before initiating flight. As a result, when they do flee, they are already closer to shelter, reducing the need for a prolonged escape. Lower field of view, which provides visual concealment from predators, is a known predictor of *S. planifrons* abundance (González-Rivero et al. 2017). The shorter fleeing distance observed in sites with a greater field of view may reflect the fact that the increased visibility allows *S. planifrons* to detect threats earlier, giving them more time to reach shelter quickly and avoid prolonged escapes. These species-specific responses may be influenced by unmeasured biotic factors such as swimming ability or visual acuity. As reef complexity continues to decline in the Caribbean, future research should explore how species-specific traits shape anti-predator responses across structural gradients.

Differences in escape responses among Stegastes species appear to be most closely linked to the field of view, as the site with the greatest field of view also exhibited the most variation in anti-predator behaviors. However, rugosity also varied across sites, with Little Bight exhibiting greater rugosity than both Sturch Bank and Coral View. Despite this variation, there were no clear differences in escape responses at Little Bight compared to the other sites. This is in contrast with previous studies that identified an inverse relationship between reef fish FID and rugosity (Chan et al. 2019; Quadros et al. 2019; Burghart et al. 2023). While both rugosity and field of view reflect elevation gradients across the reef, our results indicate that these factors are not strongly correlated. This highlights that relying solely on rugosity as a measure of structural complexity may miss ecologically relevant aspects of structure.

The anti-predator responses of wrasse and parrotfish species did not appear to differ between sites of differing complexity. Similar findings were reported by Stamoulis et al. (2019), who suggested that this lack of variation may be due to the roaming, opportunistic feeding strategies of these species. As continuous foragers, wrasse and parrotfish are constantly on the move and less reliant on specific structural features for protection or resource acquisition (Nunes et al. 2015). This mobility may reduce the influence of habitat structure on their escape responses compared to more site-attached species. Additionally, a positive correlation was found between body size and FID in both wrasse and parrotfish, supporting the asset-protection principle (Clark 1994). However, this relationship remained consistent across sites, indicating that body size influences FID consistently across sites rather than being shaped by local structural differences.

There are clear opportunities to further our understanding of how habitat complexity influences fish behavior. Large-scale 3D reconstructions provided a site-level view of reef structure, in contrast to many earlier studies that focus on more localized measurements (Nunes et al. 2015; Quadros et al. 2019). However, this broader scale introduced a spatial disconnect between the complexity metrics and the exact locations of behavioral observations. Future research could integrate both approaches, combining fine-scale complexity assessments with broader site-level measures (González-Rivero et al. 2017). Another consideration is the limitation of photogrammetry in capturing soft-bodied or dynamic elements like gorgonians and algae that move in the water. These features are common in Caribbean reefs and likely play a role in structuring habitat, yet are poorly represented in 3D models; therefore in situ methods will be necessary to assess their influence on fish behavior more accurately. Furthermore, to elicit more natural responses, we used a model grouper predator. While this method is more realistic visually compared to a diver, fish rely on a range of sensory cues, including sound, movement, and olfaction, when detecting threats (McCormick and Manassa 2008; Ladich 2019), and some influence from divers will remain inevitable (Pereira et al. 2016; Branconi et al. 2019). Future studies could further this work by incorporating multi-sensory predator cues and comparing responses to both model predators and inert objects. Together, these suggestions may help provide a more robust understanding of how structural features and predator cues interact to shape behavioral responses in reef ecosystems.

In conclusion, the relationship between structural complexity and anti-predator behavior in reef fish is species-specific. Some responses align with theories like optimal escape theory, while others do not, even among closely related species. To better understand how habitat structure shapes predator–prey interactions, it is important to consider multiple aspects of complexity across different spatial scales. This broader perspective is useful for predicting how shifts in reef structure may influence fish behavior and reshape community dynamics as reefs around the world are altered by human activities.

## Supplementary Material

araf102_Supplementary_Data

## Data Availability

Analyses reported in this article can be reproduced using the data provided by Boon et al. (2025).
