# Supplementary material for: Site-level variation in field of view is associated with altered anti-predator responses in farming damselfish
Source: Behav Ecol. 2025 Sep 12;36(5):araf102. doi: 10.1093/beheco/araf102 (PMC12477424; doi:10.1093/beheco/araf102)
Supplement: araf102_Supplementary_Data [file araf102_supplementary_data.docx]

Supplementary material


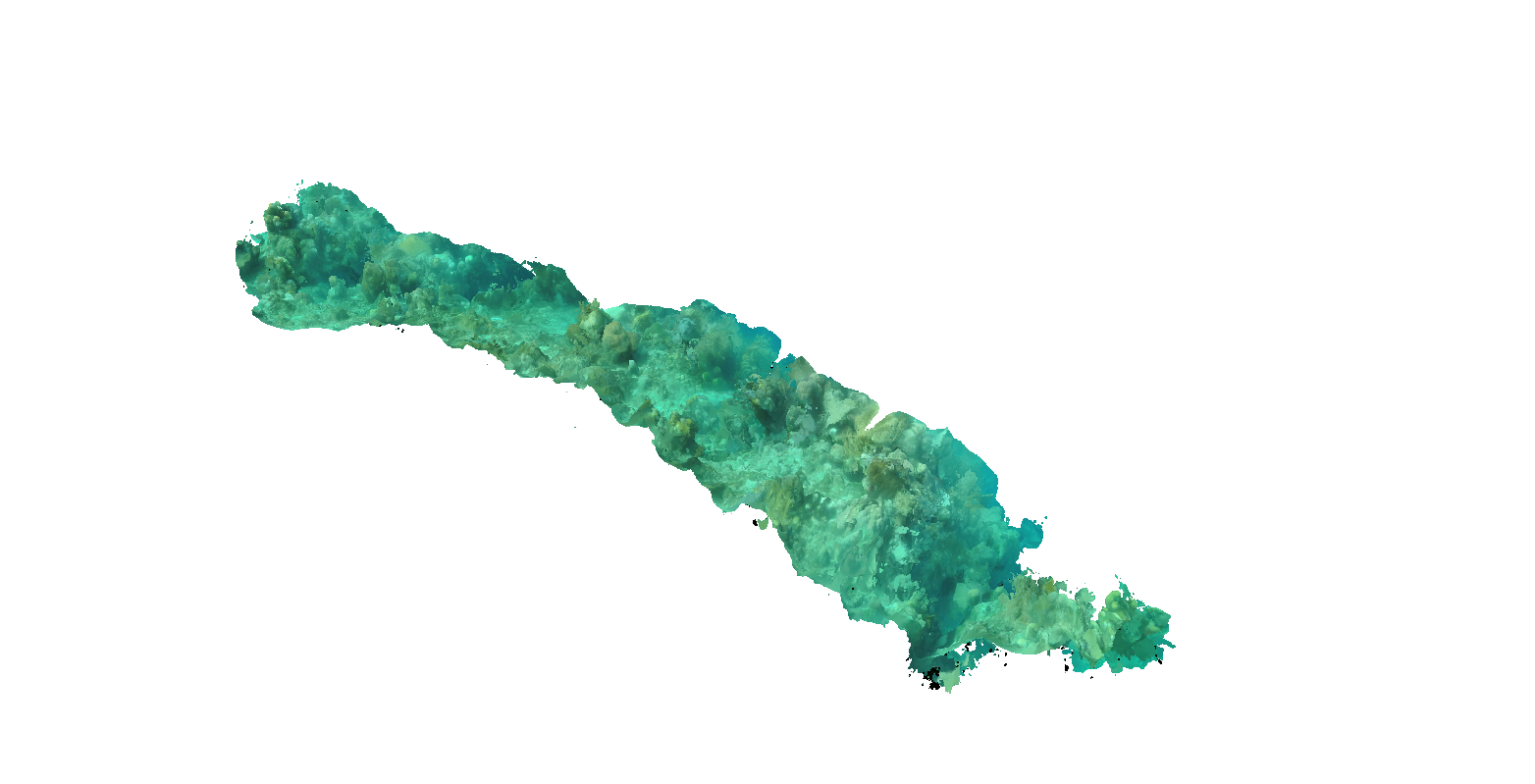

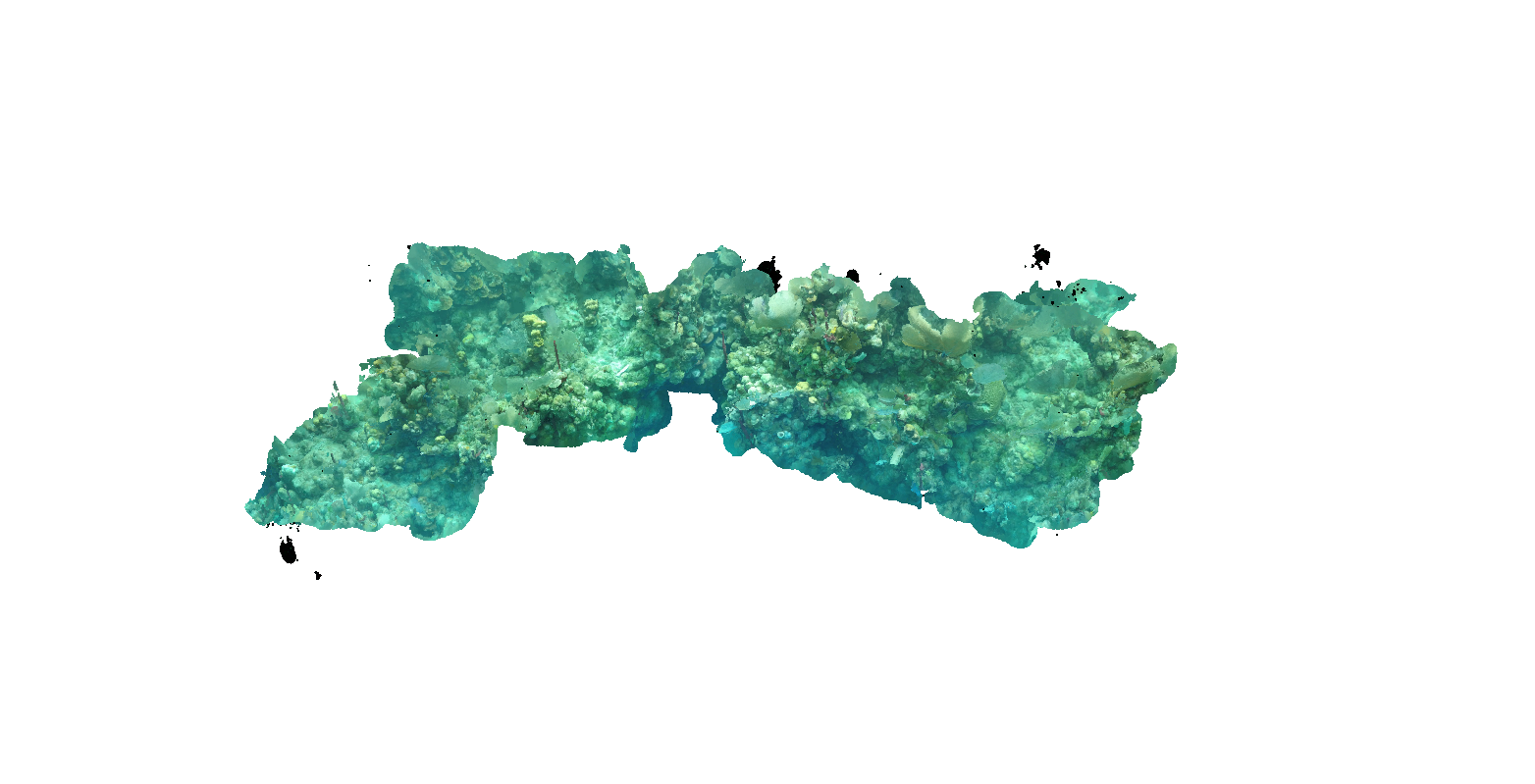

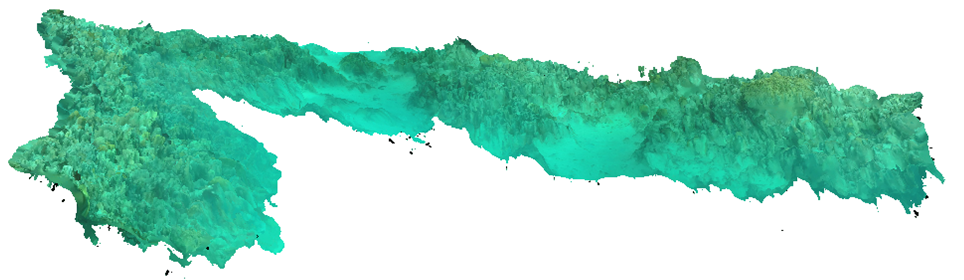


**Coral View**

**Sturch Bank**

**Little Bight**

**Figure S1** A single representative 3D model from each of the three sites (Coral View, Little Bight, and Sturch Bank).


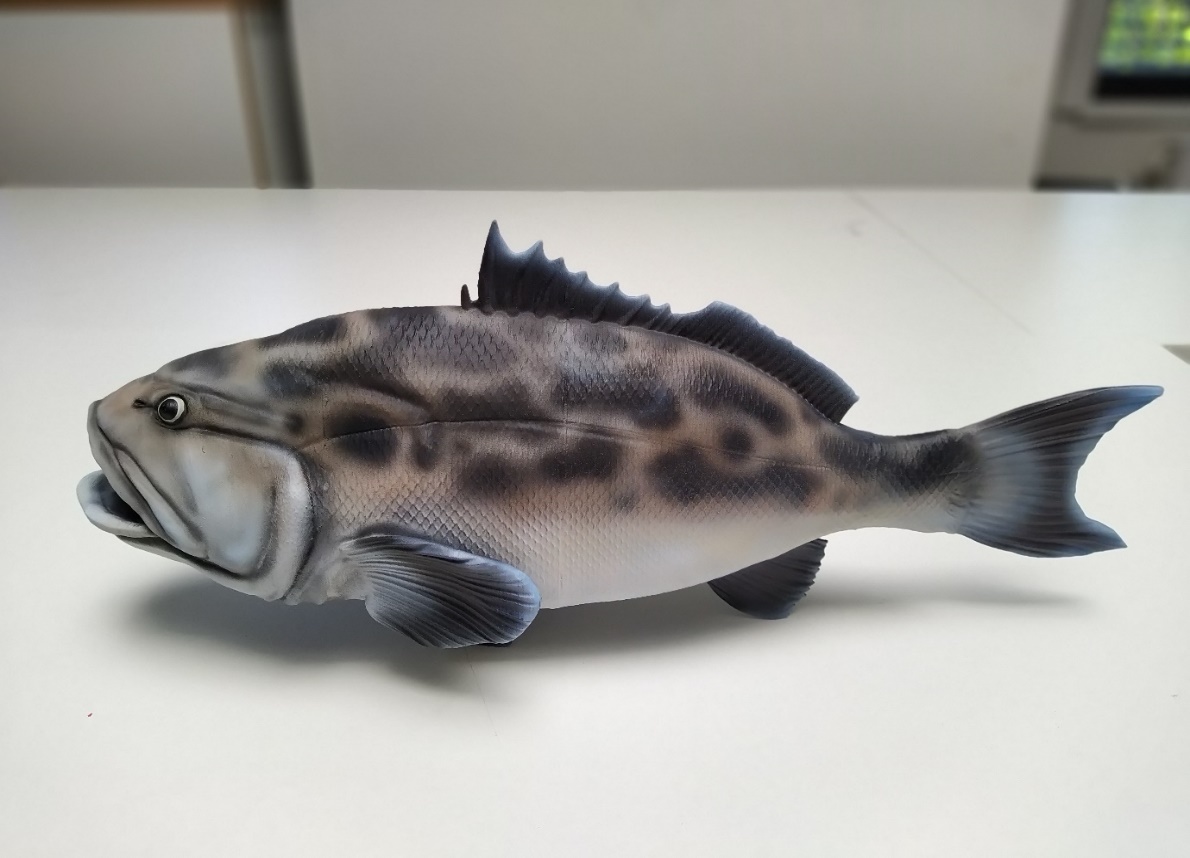


**Figure S2** Model *Mycteroperca bonaci* used in experiments. 45 cm total length. Manufactured by 3d-consultancy.com.

**Figure S3** Refuge densities across sites at sizes of **(a)** 5 cm, **(b)** 10 cm and **(c)** 15 cm depth. Violin plots illustrate the distribution of raw values, with box plots showing the median, interquartile range, and 1.5× interquartile range. Points are raw data.


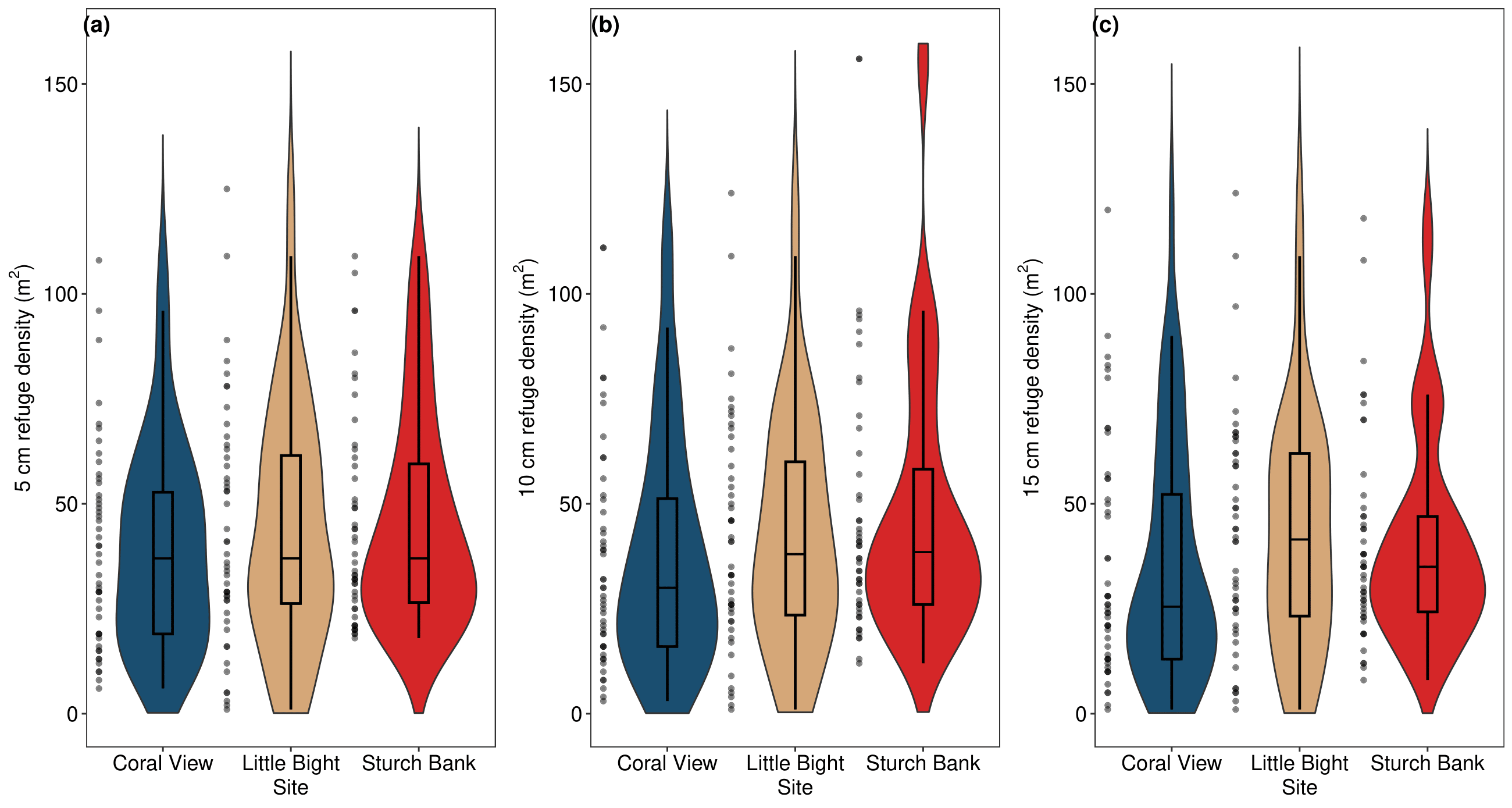

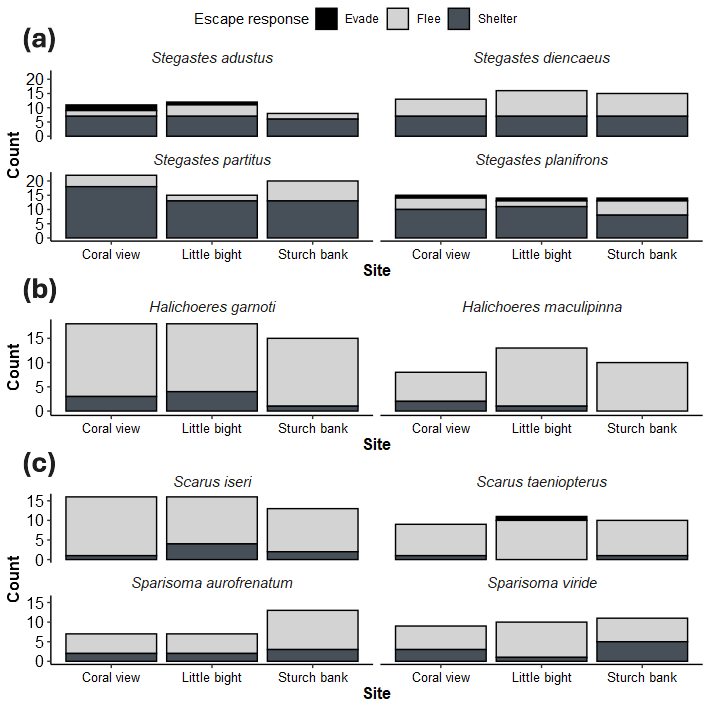


**Figure S4** Overall responses to the model predator by **(a)** damselfish, **(b)** wrasse and **(c)** parrotfish species. Each count represents a single recorded response per individual.
